# Immunosuppressants alter the immune response associated with Glucantime^®^ treatment for *Leishmania infantum* infection in a mouse model

**DOI:** 10.3389/fimmu.2023.1285943

**Published:** 2023-12-01

**Authors:** Lorena Bernardo, Jose Carlos Solana, Carmen Sánchez, Ana Torres, Eder Yaveth Reyes-Cruz, Eugenia Carrillo, Javier Moreno

**Affiliations:** ^1^ WHO Collaborating Centre for Leishmaniasis, National Center for Microbiology, Instituto de Salud Carlos III, Majadahonda, Spain; ^2^ Centro de Investigación Biomédica en Red de Enfermedades Infecciosas, Instituto de Salud Carlos III, Madrid, Spain; ^3^ LADISER Immunology and Molecular Biology, Faculty of Chemical Sciences, Universidad Veracruzana, Orizaba, Veracruz, Mexico

**Keywords:** visceral leishmaniasis, immunosuppression, immunosuppressants, BALB/c mice, TNF antagonist, methotrexate, Glucantime^®^

## Abstract

**Background:**

Immunosuppression is a major risk factor for the development of visceral leishmaniasis (VL). The number of patients receiving immunosuppressant drugs such as TNF antagonist (anti-TNF) and methotrexate (MTX) is increasing. In these patients, VL is more severe, their response to treatment poorer, and they are at higher risk of relapse, a consequence (largely) of the poor and inappropriate immune response they develop.

**Objectives:**

To examine the effect of immunosuppressive treatment on the host immune response and thus gain insight into the reduced efficacy of pentavalent antimonials in these patients. Experiments were performed using BALB/c mice immunosuppressed with anti-TNF or MTX, infected with *Leishmania infantum* promastigotes, and then treated with Glucantime^®^ at clinical doses.

**Results:**

Immunosuppression with both agents impeded parasite elimination from the spleen and bone marrow. Low pro-inflammatory cytokine production by CD4^+^ and CD8^+^ T cells was detected, along with an increase in PD-1 and IL-10 expression by B and T cells in the immunosuppressed groups after treatment.

**Conclusion:**

The immunosuppressed mice were unable to develop specific cellular immunity to the parasite, perhaps explaining the greater risk of VL relapse seen in pharmacologically immunosuppressed human patients.

## 1 Introduction

Leishmaniasis is a neglected, vector-borne, tropical disease (1). Among its clinical presentations, visceral leishmaniasis (VL) (caused by *Leishmania infantum* and *Leishmania donovani*) is the most severe form; indeed, it is fatal if patients are left untreated (2). VL affects some 0.4 million people each year worldwide (3, 4).

Immunosuppression is a major risk factor for developing VL. The effect of immunosuppression on VL has traditionally been studied in patients coinfected with HIV. Nowadays, however, VL is increasingly seen among patients receiving immunosuppressive treatment for autoimmune diseases such as rheumatoid arthritis and inflammatory bowel disease (5, 6). The Madrid (Spain) leishmaniasis outbreak of 2009 highlighted that 50% of cases of VL in immunosuppressed patients may occur in those receiving immunosuppressants (7).

The outcome of *Leishmania* infection is highly dependent on the host immune response (8). After antigen presentation and activation by dendritic cells (DCs) (9, 10), CD4^+^ and CD8^+^ T lymphocytes increase their production of pro-inflammatory Th1-type cytokines, such as IFN-γ, TNF and IL-2, to try to resolve the infection (11, 12). However, a deficient immune response leaves the infection unresolved, leading to chronic VL (13). In addition, the production of IL-6 and IL-10, two major anti-inflammatory cytokines associated with parasite persistence via the deactivation of macrophage leishmanicidal mechanisms (12), is increased. Further, this situation is associated with the expression of the programmed death cell marker PD-1, which helps drive the balance of the immune response towards the Th2-type via the reduction of pro-inflammatory cytokine production (14). Altogether, this leads to an inability to eradicate the infection.

We earlier reported that, in a mouse model of VL, short-term immunosuppressive treatment with clinical doses of TNF antagonist (anti-TNF, which blocks the TNF molecule, thus modulating the cellular immune response to *Leishmania*), or methotrexate [MTX, a folic acid antagonist which modulates folate availability to the parasite (15, 16)], could alter the immune response to *L. infantum* infection, changing the course of disease (17). Anti-TNF therapy was associated with a drastically increased liver parasite load and the inhibition of T lymphocyte functionality. MTX, however, seemed to have an antiparasitic effect, reducing the spleen parasite load. It has been suggested that MTX may exert a leishmanicidal effect via its reduction of the availability of folate, a nutrient essential for the survival of *Leishmania* (18, 19).

A further problem faced by immunosuppressed patients is their poor response to VL treatment, and consequent higher relapse rates. The *Leishmania* parasite can persist longer after treatment in such patients, leading to the reactivation of disease when immunosuppressant treatment is reintroduced (20). Successful VL treatment, which is based on the use of chemotherapeutic agents such as antimonials (e.g., meglumine antimoniate [MA]) (21), requires the host develop an adequate *Leishmania*-specific immune response. Immunosuppression may, therefore, also modify the response to VL treatment.

It has remained unknown, however, whether immunosuppressive therapy affects the response to leishmanicidal treatment with pentavalent antimonials. The aim of the present work was to characterize the course of infection in anti-TNF- and MTX-immunosuppressed BALB/c mice challenged with *Leishmania* promastigotes and treated with Glucantime^®^, recording the hosts’ antigen presentation capacity, and the humoral and cellular immune responses elicited against the parasite.

## 2 Materials and methods

### 2.1 Ethical statement

All procedures followed in this work were approved by the Committee on Ethics and Animal Welfare of the Instituto de Salud Carlos III (CBA 04_2018, PROEX 072/18), and performed according to Spanish legislation for the protection of animals for experimentation and other scientific purposes (Royal Decree 53/2013, law 32/2007), which adheres to European Directive 86/609/EEC.

### 2.2 Mice and parasites

Female BALB/c mice (7 weeks old) were purchased from Janvier-Labs (France). Promastigotes of *Leishmania infantum* strain JPC (MCAN/ES/98/LLM-724) were cultured at 26°C in RPMI-1640 L-glutamine medium (Lonza, Switzerland) supplemented with 10% foetal bovine serum (Sigma, USA), penicillin (100 U/ml) and streptomycin (100 µg/ml) (Lonza) (hereafter RPMIc medium).

### 2.3 Immunosuppression, infection, and treatment

**Figure 1:**
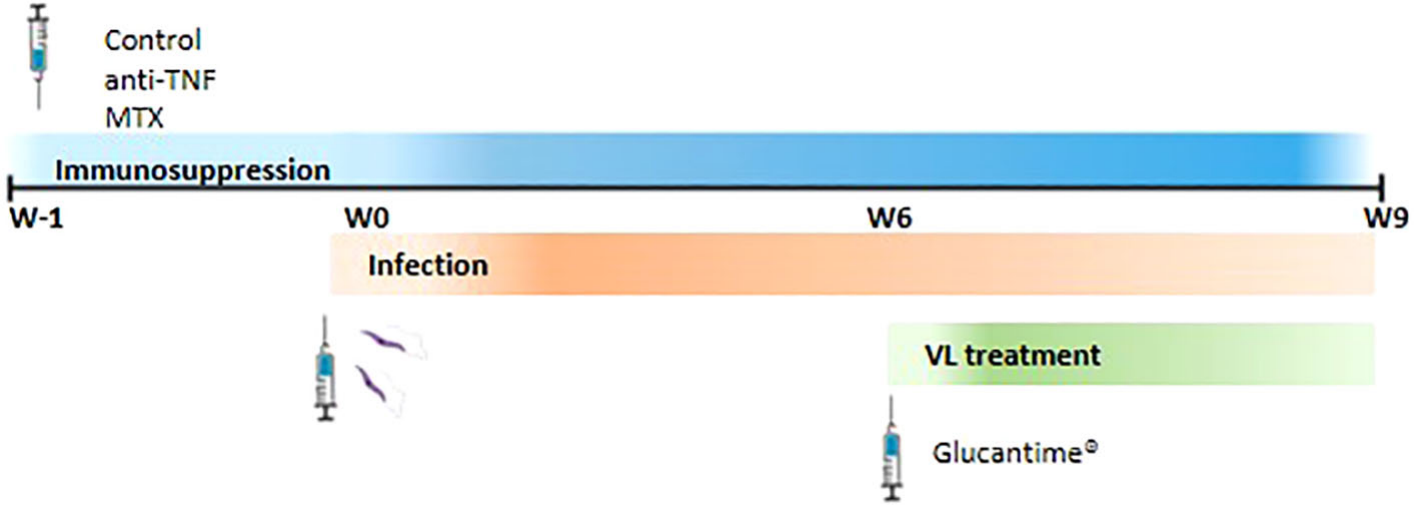
Representation of the experimental design. BALB/c mice were divided into three groups and they were i.p. administrated with PBS three times per week (control group), 20 mg/kg anti-TNF twice per week or 2.5 mg/kg MTX three times per week. These regiments were maintenance until the end of the experiment. One week after (W0) mice were intravenously infected with *L. infantum* promastigotes. Six weeks post-infection, animals were i.p. treated with 20 mg/kg Glucantime^®^ per day for 21 days (W9).

Mice were divided into three groups (n=6 per group) and received either: 1) intraperitoneal (i.p.) PBS 1X (Gibco, USA) three times per week (control group); 2) i.p. anti-TNF antibodies 20 mg/kg (Leinco Technologies, USA) twice per week (22); or 3) i.p. MTX 2.5 mg/kg (Sigma, USA) three times per week (23), all for up to nine weeks (W9). After one week of immunosuppression (W0), the mice were intravenously infected via the tail vein with 1 x 10^7^ stationary phase promastigotes in 200 µl PBS (**Figure 1**). At six weeks post-infection (W6) they were treated with i.p.-MA (Glucantime^®^) (Sanofi, France) at 20 mg/kg per day - the recommended clinical dose (21) - for 21 days (W9). All treatments were administrated in 0.1 ml volumes.

### 2.4 Circulating T and B lymphocyte populations as determined by flow cytometry

At W1, W6 and W9, 50 µl of blood were extracted from the submaxillary vein into an EDTA-containing tube and used to determine the number of circulating cells employing a Scill Vet ABC Plus automatic counter (Horiba Medical, Japan). To determine T and B lymphocytes numbers, erythrocytes were eliminated using ammonium-chloride-potassium (ACK) lysis buffer (150 mM NH4Cl, 10 mM KHCO3 and 0.1 mM EDTA at pH 7.2) for 20 min. Centrifugation at 800 g for 6 min with PBS + 2% bovine serum albumin (BSA) was then performed to wash the cells. APC CD3 (Clone 17A2, Biolegend, USA), PE-Cy5 CD4 (Clone GK1.5, eBioscience, USA), PE CD8 (Clone 53-6.7, Biolegend) and FITC CD19 (Clone MB19-1, Biolegend) anti-mouse antibodies were used to stain CD3^+^, CD4^+^, CD8^+^ T cells and CD19^+^ B cells, respectively, for 30 min at 4°C in the dark. After washing with PBS + 2% BSA, the stained cells were fixed in PBS-2% formaldehyde. Cell populations were determined using a BD Accuri C6 Plus flow cytometer (Beckton Dickinson Biosciences, USA) and employing FlowJo v7.6.5 software (FlowJo LLC, USA). Total T or B cells/mm^3^ were obtained by multiplying the frequency of each population by the number of leukocytes determined in the blood sample.

### 2.5 Parasite load quantification by qPCR

Animals were euthanized at W6 or W9. After necropsy, liver, spleen and bone marrow (BM) samples were taken from each animal and the number of *L. infantum* parasites determined by quantitative real-time PCR (qPCR). Samples were processed as previously described (17). Briefly, liver and spleen tissues were homogenized in RPMIc medium using a 40 µM Falcon Cell Strainer (Thermo Fisher Scientific, USA) and centrifuged for 5 min at 800 g. The resulting cell suspension was then lysed with ACK lysis buffer to eliminate erythrocytes. After washing, the suspension was resuspended in 4 ml (liver) or 2 ml (spleen) RPMI medium supplemented with L-glutamine, 10% FBS, non-essential amino acids (0.44 mM L-alanine, 0.4 mM L-asparagine, 0.343 mM L-glutamate), penicillin (100 U/ml), streptomycin (100 µg/ml), 50 µg/ml gentamicin and 10 µM β-mercaptoethanol (all from Sigma, USA). BM was obtained from the femur by flushing PBS into the bone’s central cavity. The cell suspension collected was suspended in 1 ml of RPMI supplemented as described above. DNA was then extracted using the phenol:chloroform:isoamyl alcochol (25:24:1) method (24), and resuspended in 100 µl of distilled water. The DNA concentration was measured using a NanoDrop One spectrophotometer (Thermo Fisher Scientific). Parasite loads were then determined by qPCR using the LightCycler FastStart Kit (Roche Applied Science, Germany), employing the primers R223 and R334 for the *Leishmania* 18S ribosomal subunit (25). To calculate the parasite concentration (parasites/µl) of each sample, a standard curve was used as previously described (26). The Figs obtained were then divided by the DNA concentration of the tissue to provide values for parasites/µg DNA, and then multiplied by the total number of µg in the initial volume in which each sample was resuspended, providing the total number of parasites (performed per organ).

### 2.6 Humoral immune response was determined by ELISA

Serum preparations were made at W1, W6 and W9 from 200 µl of blood extracted from the submaxillary vein. These samples were coagulated after incubation at 37°C for 30 min followed by 4°C overnight, and then centrifuged at 17,000 g for 15 min. The isolated sera were maintained at -20°C until the determination of antibody titres against *Leishmania* by ELISA. Briefly, Nunc MaxiSorp plates (Thermo Fisher Scientific) were coated overnight at 4°C with 100 µl of 2 µg/ml soluble *L. infantum* antigen (SLA) obtained as previously described (27). They were then washed with PBS-0.5% Tween20 and blocked with 3% BSA in PBS-Tween20 (blocking solution) for 1 h at 37°C. A 1/2 serial dilution of these samples was then made and incubated for 1 h at 37°C. The wells were then washed and incubated for 30 min at 37°C with the horseradish peroxidase-conjugated secondary antibodies anti-IgG, anti-IgG1 and anti-IgG2a (Nordic Mubio, Netherlands), diluted 1:4000 in blocking solution for IgG and IgG2a, and 1:2000 for IgG1. After a final wash, the reaction was allowed to proceed using the SIGMAFAST OPD Kit (Sigma, USA), following the manufacturer’s instructions. The colorimetric reaction was stopped by the addition of 2N HCl, and the absorbance measured at 490 nm in a MultiskanFC device (Thermo Fisher Scientific). Antibody titres were calculated as the inverse of the last serial dilution value that was greater than that of the negative control.

### 2.7 Cytokine concentrations in supernatants

For cytokine analysis, 1 x 10^6^ splenocytes were placed in each well of a high-binding 96-well plate and stimulated in 200 µl volumes with plate-bound αCD3 (10 µg/ml) plus soluble αCD28 antibodies (2 µg/ml), or with RPMIc (non-stimulated control), for 24 h in a 5% CO_2_ atmosphere at 37°C. Supernatants were collected and the IFN-γ, IL-2, IL-4, IL-6, TNF, IL-17A and IL-10 concentrations measured using the BD Cytometric Bead Array Mouse Th1/Th2/Th17 Cytokine Kit (Beckton Dickinson Biosciences) following the manufacturer’s recommendations with some modifications. Briefly, 50 µl of each sample were plated with 50 µl of a mixture of the beads and 50 µl of PE-detection reagent. The samples were then incubated for 2 h at room temperature before centrifuging at 400 g for 8 min. The pellets were each resuspended in 150 µl of wash solution. A standard curve was then constructed. Cytokine concentration data were acquired using a BD Accuri C6 Plus flow cytometer (Beckton Dickinson Biosciences), and analyzed using FlowJo v.7.6.5 software (FlowJo LLC).

### 2.8 Flow cytometry analysis of splenocyte populations

1 x 10^6^ splenocytes were stimulated in a 96-well plate with 200 µl of 10 µg/ml *L. infantum* SLA for 72 h, along with a αCD3/αCD28 cocktail or RPMIc (non-stimulated control) for 24 h at 37°C in a 5% CO_2_ atmosphere. During the last 6 h of culture, 10 µg/ml of brefeldin A (Sigma, USA), were added to block the secretion of cytokines. Once the incubation was finished, the splenocytes were centrifuged at 800 g for 2 min and washed with 150 µl of PBS with 1% FBS (FACS solution). The cells were then incubated for 5 min at 4°C with mouse FcBlock (BD Bioscience, USA and) diluted 1:100 in FACS solution. Surface antigens were then detected using different antibodies diluted 1:400, incubating for a further 20 min. For the populations that required surface antigen staining only, and for the detection of the exhaustion PD-1 marker, the cells were washed with FACS solution, fixed with 2% formaldehyde in PBS for 15 min, and resuspended in 200 µl of FACS solution. The T cells were stained with PerCp-Cy5 anti-mouse CD3e (Clone 145-2C11), BUV395 anti-mouse CD4 (Clone GK1.5) and FITC anti-mouse CD8 (Clone 53-6.7); B cells were stained using a BV510 anti-mouse CD19 (Clone ID3) antibody. For DCs, the antibodies used were BV785 anti-mouse Ly6C (Clone HK1.14), AlexaFluor 647 anti-mouse Ly6G (Clone 1A8), APCH7 anti-mouse CD11c (Clone N418), PECy7 anti-mouse CD11b (Clone M1/70) and FITC anti-mouse MHCII (Clone 39-10-8). For the detection of the PD-1 exhaustion marker, a BV711 anti-mouse PD-1 (Clone 29F.1A12) antibody was used. The DC population was characterized as follows: conventional dendritic cells (cDCs) CD8^-^ (CD11b^+^, CD11c^+^, MHCII^+^, Ly6C^-^, Ly6G^-^), cDCs CD8^+^ (CD11b^-^, CD11c^+^, MHCII^+^, Ly6C^-^, Ly6G^-^), plasmacytoid dendritic cells (pDCs) (CD11b^-^, CD11c^+^, MHCII^lo^, LY6C^+^, Ly6G^-^) and inflammatory DCs (Tip-DCs) (CD11b^+^, CD11c^+^, MHCII^+^, Ly6C^+^). For populations that required intracellular staining, excess surface antibodies were first washed away and the cells fixed and permeabilized with BD Cytofix/Cytoperm solution for 20 min at 4°C, and then washed with PermWash buffer (BD, USA). A 1:100 dilution in PermWash buffer was then made to stain intracellular cytokines for 30 min at 4°C with PECy7 anti-mouse IFN-γ (Clone XMG1.2), PE anti-mouse IL-2 (Clone JES6-5H4), alexaFluor 647 anti-mouse TNF (Clone MP6-XT22), BV421 anti-mouse IL-10 (Clone JES5-16E3) or with PE anti-mouse iNOS (Clone W16030C). Finally, cells were washed twice with PermWash and resuspended in 200 µl of FACS solution. The frequency of cytokine-producing T cells was determined using a BD LSRFortessa X-20 flow cytometer (BD Biosciences, USA), employing a Boolean gating routine performed with FlowJo v.7.6.5 software.

### 2.9 Statistical analysis

When data were normally distributed according to the Shapiro-Wilk test, they were examined via the two-tailed Student t test; when they were not, they were examined via the Mann-Whitney U test. Significance was set at p<0.05. All statistical analyses were performed using Graph-Pad Prism 9 software (USA).

## 3 Results

### 3.1 The outcome of Glucantime^®^ treatment is affected by the immunosuppressants anti-TNF and MTX

**Figure 2:**
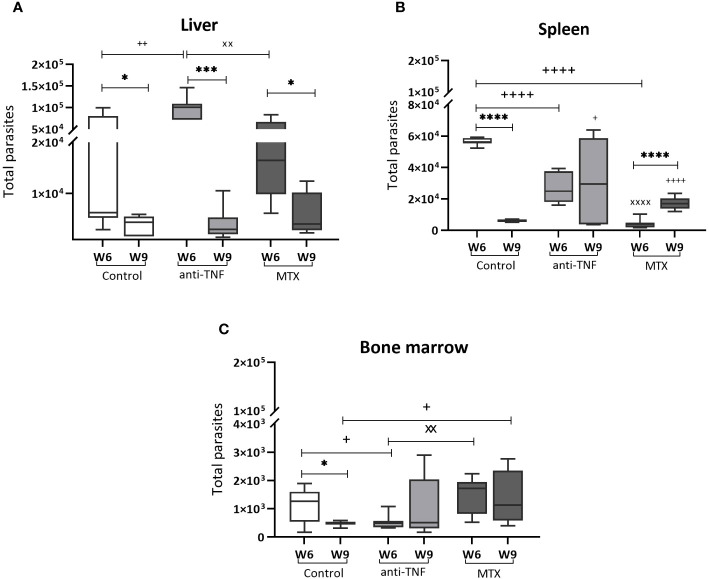
Parasite load in VL target organs before and after Glucantime^®^ treatment. BALB/c mice were immunosuppressed with anti-TNF, MTX or treated with PBS and infected one week later with 1 x 10^7^ *L. infantum* promastigotes. At 6 weeks post-infection (W6), Glucantime^®^ treatment was administered for 21 days (until W9). Animals were euthanized both at W6 and W9, and the total parasite load determined by qPCR in the liver **(A)**, spleen **(B)** and bone marrow **(C)**. The results are presented are medians and whisker (min to max) plots for each group. Significant differences within groups at W6 and W9 are marked with a * sign; a ^+^ sign indicates significant differences compared to the control group for the same week; a ^x^ sign indicates differences between immunosuppressed groups at W6 or W9 (p<0.05). ***p<0.001; ****p<0.0001; ^++^p<0.01; ^++++^p<0.0001; ^xx^p<0.01; ^xxxx^p<0.0001.

At W6, the anti-TNF-treated mice had a significantly higher liver parasite load than the control animals (**Figure 2A**). After Glucantime^®^ treatment (W9), a significant reduction in the parasite load was observed in all groups, with no differences among them. At W6, the parasite load in the spleen of the control group animals had significantly increased (5.65 x 10^4^ total parasites) compared to anti-TNF and MTX-treated animals (**Figure 2B**). At this time, however, the MTX-treated animals showed the lowest spleen parasite load of all groups (0.41 x 10^4^ total parasites). Interestingly, Glucantime^®^ treatment was only effective in reducing parasite numbers in the spleen of the control group (p<0.0001). The total number of spleen parasites before and after Glucantime^®^ treatment did not change with the anti-TNF treatment, suggesting an impairment of parasite control by this immunosuppressant. However, a significant increase was seen in the spleen of the MTX group members (1.71 x 10^4^ total parasites; p<0.0001).

In the bone marrow, Glucantime^®^ was effective in reducing the parasite load in the control group only (0.47 x 10^3^ total parasites at W9) (**Figure 2C**). In the MTX group, the parasite load at W9 was significantly higher than in the control group (1.3 x 10^3^ total parasites; p=0.039), as MTX prevented parasite elimination, and higher numbers were observed between W6 and W9 in this group.

These results showed that anti-TNF and MTX influence VL treatment with Glucantime^®^ by affecting parasite load reduction, particularly in the spleen and bone marrow. It should be noted that the potential antiparasitic effect of MTX seen at W6, appeared to be reversed after antimonial treatment.

### 3.2 Anti-TNF generated marked hypergammaglobulinemia after Glucantime^®^ treatment

**Figure 3:**
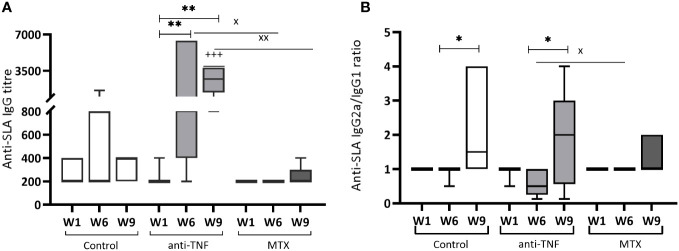
Humoral immune response of immunosuppressed mice to *Leishmania* soluble antigen before and after Glucantime^®^ treatment. Sera from BALB/c mice immunosuppressed with anti-TNF, MTX or PBS and infected with *L. infantum*, were collected one week after infection (W1), before Glucantime^®^ treatment (W6), and after Glucantime^®^ treatment (W9), to determine anti-SLA specific antibody titres by ELISA. The results are shown as median and whisker plots for anti-SLA IgG antibody titres **(A)** and the anti-SLA IgG2a/IgG1 ratio **(B)**. Significant differences within groups at W6 and W9 are marked with a * sign; a ^+^ sign indicates significant differences compared to the control group for the same week; a ^x^ sign indicates differences between immunosuppressed groups at W6 or W9 (p<0.05). **p<0.01; ^+++^p<0.001; ^xx^p<0.01

In the anti-TNF-treated animals, the titre of IgG antibodies to *Leishmania* antigens increased significantly (p=0.0087) by W1, reaching the highest values for all groups at W6 (**Figure 3A**). Although Glucantime^®^ treatment reduced this titre at W9, it continued to be higher than that recorded for the control (p=0.0006) and MTX groups (p=0.0043). IgG production was almost absent in the MTX-treated animals. In addition, Glucantime^®^ treatment increased the IgG2a/IgG1 profile in all groups (**Figure 3B**). Importantly, the antiparasitic treatment inverted the polarization towards IgG1-type antibodies observed at W6 in the anti-TNF group. Although the low *Leishmania*-specific total IgG-titre in the MTX-treated animals was low (these animals showed very little reactivity from W1 onwards) (**Figure 3A**), a polarization towards the IgG2a subclass was observed (**Figure 3B**).

Glucantime^®^ treatment resulted in the increase of the relative abundance of the IgG2 subtype in all the groups studied, including the anti-TNF group, in which IgG subtype polarization was reversed. Antiparasitic treatment was also able to reduce the parasite-specific IgG titter in this group.

### 3.3 Anti-TNF and MTX affect the number of circulating lymphocytes after Glucantime^®^ treatment

**Figure 4:**
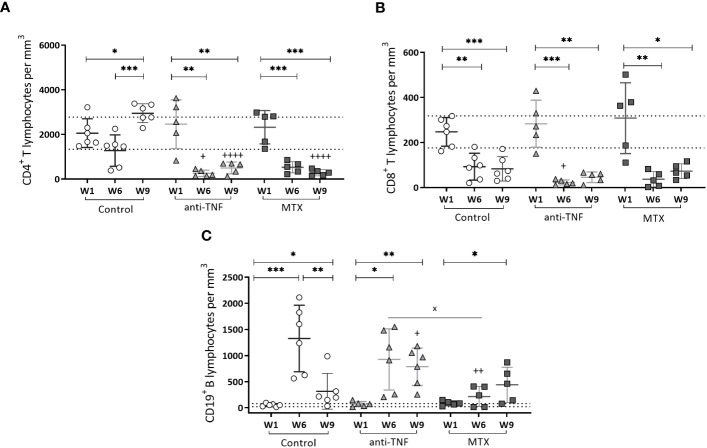
Circulating T CD4^+^, T CD8^+^ and B CD19^+^ cells in peripheral blood of immunosuppressed mice. T CD4^+^ **(A)**, T CD8^+^ **(B)** and B CD19^+^ **(C)** cells were analyzed by flow cytometry. Results are shown as the mean ± standard deviation for each group at W1, W6 and W9. The dotted lines show the normal values for each population: T CD4 + 2059.38 ± 720.18/mm^3^; T CD8 + 247.11 ± 70.94/mm^3^ and B CD19 + 51.13 ± 30.70/mm^3^ determined in-house in naïve mice of the same age. Significant differences within groups at W6 and W9 are marked with a * sign; a ^+^ sign indicates significant differences compared to the control group for the same week; a ^x^ sign indicates differences between immunosuppressed groups at W6 or W9 (p<0.05) **p<0.01; ***p<0.001; ^++^p<0.01; ^++++^p<0.0001.

At W1, no differences were detected among the groups in terms of the number of circulating CD4^+^ T (**Figure 4A**), CD8^+^ T (**Figure 4B**) and CD19^+^ B (**Figure 4C**) cells; all values remained within the range of naïve mice of the same age. At W6, the number of CD4^+^ T cells was drastically reduced to below normal values in both immunosuppressed groups (**Figure 4A**). After the antimonial treatment, normal values were only recovered in the control group; in the immunosuppressed animals they remained below normal limits.

A significant reduction in CD8^+^ T circulating cells was seen for all immunosuppressed groups at W6 compared to the control group, particularly so in the anti-TNF-treated mice (p=0.030). Despite the recovery of circulating CD4^+^ T cells, normal CD8^+^ T lymphocytes levels did not recover after Glucantime^®^ treatment in any group (**Figure 4B**).

Finally, during the development of chronic VL infection (W6), a significant increase was seen in the number of CD19^+^ B cells in all groups, exceeding normal values (**Figure 4C**). At that time of infection, these cells were more numerous in the anti-TNF group than in the MTX-treated animals (p=0.029). Once the antimonial treatment was finished at W9, a reduction in the CD19^+^ B population was observed in the control group, but not in the immunosuppressed animals (values were higher than in the control group; p=0.041).

The findings revealed that the restoration of CD4^+^ T-cell and CD19^+^ B-cell populations to normal levels in the bloodstream of BALB/c mice following antimonial treatment is affected by the immunosuppressive effects of both anti-TNF and MTX.

### 3.4 Immunosuppressant treatment increases cell exhaustion and IL-10 after antimonial treatment

**Figure 5:**
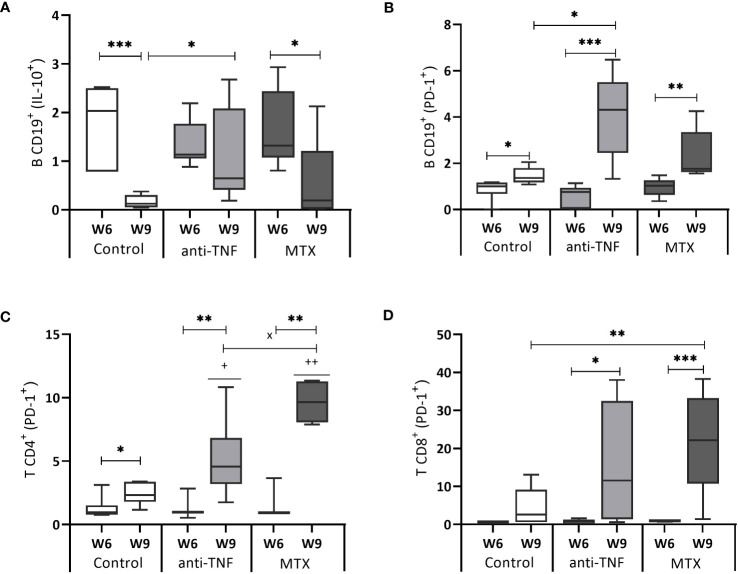
IL-10 and PD-1 expression by B CD19^+^, T CD4^+^ and T CD8^+^ cells. After 72 h of SLA stimulation, splenocytes were stained and analyzed by flow cytometry to determine the frequency of B CD19^+^ cells producing IL-10 **(A)**, as well as the expression of the cell exhaustion marker PD-1 by B CD19^+^ cells **(B)**, T CD4^+^ cells **(C)** and T CD8^+^ cells **(D)**. The results are expressed as the SLA/RPMI ratio, and shown as median and whisker plots. Significant differences within groups at W6 and W9 are marked with a * sign; a ^+^ sign indicates significant differences compared to the control group for the same week; a ^x^ sign indicates differences between immunosuppressed groups at W6 or W9 (p<0.05) **p<0.01; ***p<0.001; ^++^p<0.01.

After Glucantime^®^ treatment (W9), the control (p=0.0006) and MTX groups (p=0.036) showed a significant reduction in the spleen IL-10^+^ CD19^+^ B population. In the anti-TNF-treated animals, the frequency of IL-10-producing-CD19^+^ B cells at W9 was higher than in the control group (p=0.015) (**Figure 5A**). The CD19^+^ B population of the same animals showed the highest frequency of cells expressing the PD-1 exhaustion marker following Glucantime^®^ treatment (**Figure 5B**) (p=0.015 compared to the control group) (**Figure 5B**). Similarly, the immunosuppressed mice showed higher frequencies of CD4^+^ and CD8^+^ T lymphocytes expressing PD-1^+^ at W9 (**Figures 5C, D**). Regarding the frequencies of Treg (CD44^hi^ CD127^-^ CD25^+^) population, no statistical differences were found between groups at W6 or W9 (data not shown).

Both immunosuppressants increased the production of PD-1 and IL-10, leading to a shift in the protective immune response after VL treatment toward a susceptible profile.

### 3.5 Anti-TNF and MTX immunosuppression altered the Th1-type immune response generated after Glucantime^®^ treatment

**Figure 6:**
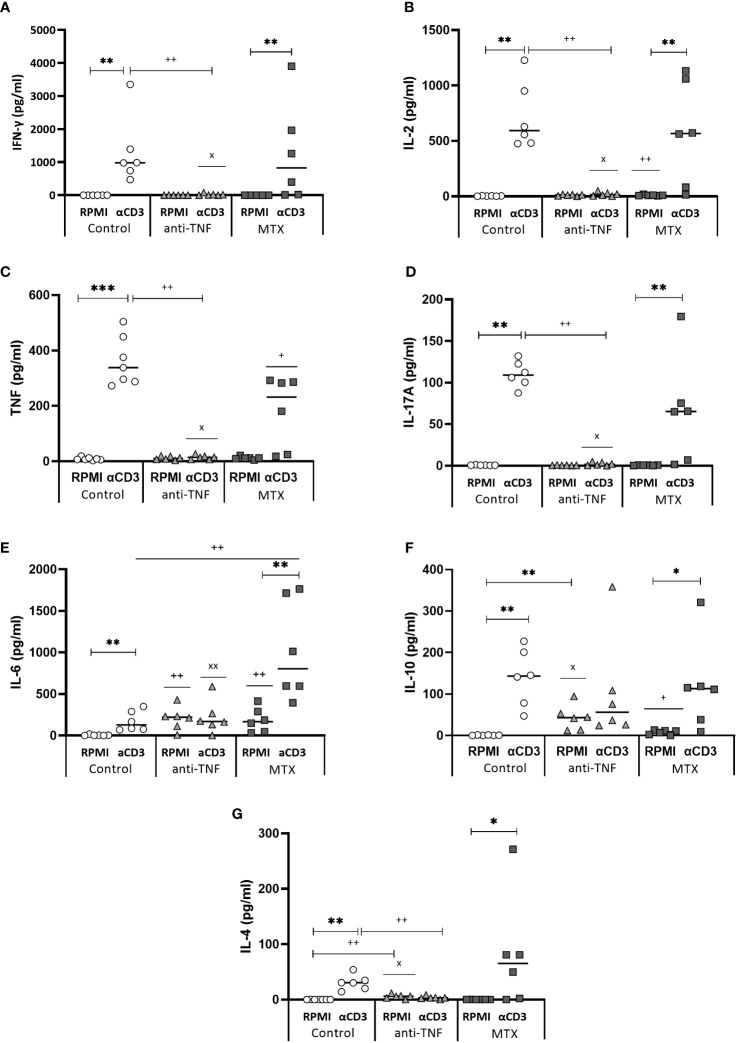
Cytokine production after splenocyte stimulation with αCD3/αCD28. At W9, after the Glucantime^®^ treatment, splenocytes were stimulated with αCD3/αCD28 for 24 h. The supernatants were collected and IFN-γ **(A)**, IL-2 **(B)**, TNF **(C)**, IL-17 **(D)**, IL-6 **(E)**, IL-10 **(F)** and IL-4 **(G)** cytokines secreted to the medium analysed using the CBA BD Th1/Th2/Th17 Kit. The Mann-Whitney test was used to analyse the differences between groups (p<0.05). Significant differences within groups at W6 and W9 are marked with a * sign; a ^+^ sign indicates significant differences compared to the control group for the same week; a ^x^ sign indicates differences between immunosuppressed groups at W6 or W9 (p<0.05) **p<0.01; ^++^p<0.01; ^xx^p<0.01.

After stimulation of the splenocytes with a non-specific αCD3/αCD28 cocktail, differences were seen between the immunosuppressed groups in terms of the cytokines released to the medium (**Figure 6**). Splenocytes from the anti-TNF-treated mice did not secret any of the pro-inflammatory cytokines (IFN-γ, IL-2 and TNF); consequently there was no Th1-type response in these animals (**Figures 6A–C**). No stimulation of IL-17 production was observed in this group either (**Figure 6D**). In the MTX-treated animals, αCD3/αCD28 stimulation generated concentrations of the pro-inflammatory cytokines IFN-γ and IL-2 similar to those observed in the control group, although the TNF level was significantly reduced (p=0.026). With regard to the production of cytokines related to the susceptibility to progress towards VL (IL-6, IL-10 and IL-4), IL-6 production in the MTX-treated animals was considerably higher than in the control (p=0.037) and anti-TNF groups (p=0.018) (**Figure 6E**). Interestingly, the basal levels (in RPMI) of IL-6 (**Figure 6E**) and IL-10 (**Figure 6F**) in the anti-TNF-treated animals were already as high as those obtained after αCD3/αCD28 stimulation, and indeed higher than in both other groups, suggesting that both cytokines are constitutively produced under anti-TNF treatment. The control and MTX groups showed reduced IL-4 production under anti-TNF immunosuppression (**Figure 6G**).

**Figure 7:**
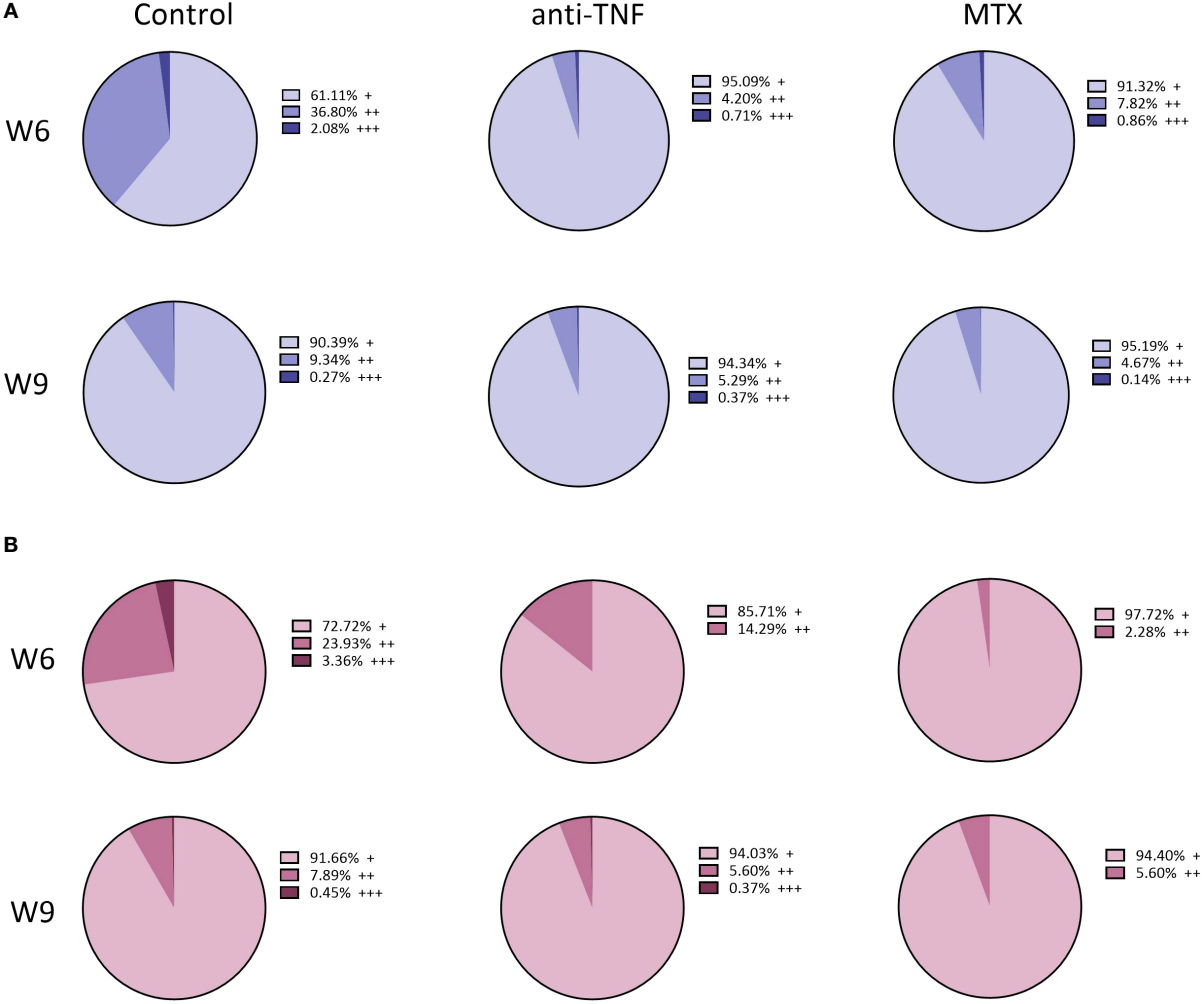
Total percentage of T-producing lymphocytes in the spleen of immunosuppressed mice, following SLA stimulation. Immunosuppressed and infected BALB/c mice were euthanized before (W6) and after (W9) Glucantime^®^ treatment. The splenocytes were stimulated for 72 h with *L. infantum* SLA to determine, by flow cytometry, the cytokine-producing capacity of the T CD4^+^ and T CD8^+^ cells via a Boolean gating analysis. Results are shown as the total percentage of single- (+), double- (++) and triple- (+++) cytokine-producing CD4^+^ T **(A)** and CD8^+^ T **(B)** among T lymphocytes, before and after antimonial treatment.

After determining the capacity of the T cells to produce these cytokines after non-specific stimulation, their capacity to produce pro-inflammatory cytokines in response to specific SLA stimulation was examined. The percentage of single producer-CD4^+^ T cells increased only in the control group. No differences in double and triple producer-CD4^+^ T cells were seen between groups at W9. Among the CD4^+^ T cells that produced any of the examined cytokines at W6, the frequency of double and triple producers for the control group was 36.80% and 2.08%, respectively; in the immunosuppressed animals, however, these percentages were much lower (**Figure 7**). In the anti-TNF-immunosuppressed animals, only 4.20% of CD4^+^ T lymphocytes produced two cytokines, and 0.71% produced three at the same time, while in the MTX group these percentages were 7.82% and 0.86%, respectively (**Figure 7A**). Only 3.36% of CD8^+^ T lymphocytes from the control group were able to produce the three pro-inflammatory cytokines at the same time at W6 (**Figure 7B**). No triple-producers were observed in the MTX-treated animals before or after antimonial treatment. The frequencies of double and triple producer-CD8^+^ T cells observed at W9 in the anti-TNF group were much lower than in the control animals.

The results after TCR stimulaton indicated that Th1 expansion is suppressed by anti-TNF administration, as evidenced by reduced production of production of IFN-γ, IL-2 and TNF, besides a significant basal level of IL-10. The lower ratio IFN/IL-10 ratio, in conjunction with elevated antibody titers, may indicate also an expansion of Th2 cells, although no IL-4 was detected. On the other hand, MTX appeared to favor the expansion of Th17 (IL-6, IL-17A) and also Th2 cells (IL-4). *Leishmania-*specific cellular response revealed that both anti-TNF and MTX immunosuppressants decreased the production of pro-inflammatory cytokines by multifunctional cells, suggesting a discrete anti-*Leishmania* Th1-type immune response after Glucantime^®^ treatment.

### 3.6 Immunosuppression affected the dendritic cell populations after antimonial treatment

**Figure 8:**
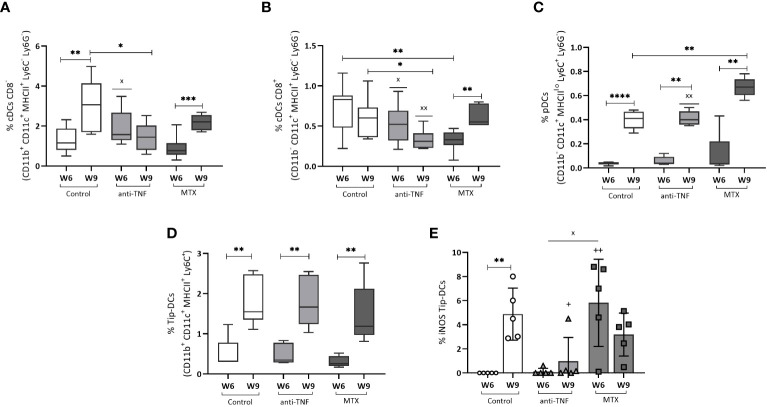
Dendritic cell populations before and after Glucantime^®^ treatment. Splenocytes were analyzed *ex vivo* by flow cytometry to examine the dendritic cell populations in the spleen: cDCs CD8^-^ **(A)**, cDCs CD8^+^ **(B)** and pDCs **(C)**. Stimulation with αCD3/αCD28 for 24 h was allowed to determine the frequency of Tip-DCs **(D)** and NO production by Tip-DCs **(E)**. Differences between groups in all the DCs populations were examined using the Student t test: the Mann-Whitney test was used to determine differences in NO production (p<0.05). Significant differences within groups at W6 and W9 are marked with a * sign; a ^+^ sign indicates significant differences compared to the control group for the same week; a ^x^ sign indicates differences between immunosuppressed groups at W6 or W9 (p<0.05) **p<0.01; ****p<0.0001; ^xx^p<0.01; ^++^p<0.01.

A significant increase in the frequency of cDCs CD8^-^ in the control and MTX groups was recorded after Glucantime^®^ treatment (3.06% p=0.0052, and 2.17% p=0.0009, respectively) (**Figure 8A**). This cDCs CD8^-^ population was smaller in the anti-TNF-treated animals than in the control group (p=0.016). The cDCs CD8^+^ population was smaller in the anti-TNF group than in all other groups (**Figure 8B**). In contrast, pDCs were increased after antimonial treatment in all animals (**Figure 8C**), very significantly so in the MTX group compared to the control (p=0.0043). After αCD3/αCD28 stimulation, an increase was detected in the frequency of TipDCs in all groups at W9 (**Figure 8D**). In the control group this increase was associated with higher iNOs production (**Figure 8E**); in the anti-TNF-treated animals these cells produced less iNOs than did the controls (p=0.0079). Although the MTX animals showed higher NO production at W6 than did anti-TNF and control groups, no increase was seen after antimonial treatment.

Analysis of distinct DC populations revealed that anti-*Leishmania* treatment results in an increase in the frequency of various cell subsets. However, continuous administration of anti-TNF reduced the proportion of conventional dendritic cells (cDCs), potentially leading to diminished antigen presentation at the end of VL treatment.

## 4 Discussion

Immunosuppressive treatments, such as anti-TNF and MTX, clearly increase the risk of developing VL, by altering the cellular immune response against the parasite, as we had previously demonstrated (17). The present results suggest that treatment for VL with pentavalent antimonials is effective in reducing parasite loads in the liver, even under immunosuppression with anti-TNF or MTX. However, the spleen parasite load was not reduced in animals immunosuppressed by these agents (unlike in the controls), leading to poorer treatment outcomes. It is not known if this also happens in immunosuppressed patients undergoing these treatments since parasite detection is not routinely performed after treatment if symptoms disappear (and even if they do, analyses are performed on blood). However, it is well known that relapses occur more commonly in immunosuppressed patients, especially in those treated with anti-TNF (20).

Interestingly, the present results showed a reduced parasite load in the spleen of the MTX-treated animals before treatment with antimonials, as previously described (17). MTX thus appears to have a leishmanicidal effect, probably because it reduces the levels of folate and pteridine, which are needed for parasite growth (28). However, this potential leishmanicidal effect at W6 was altered by W9, with the number of parasites in the spleen higher than in the control group. The larger post-antimonial parasite numbers in the spleen, and also in the bone marrow, of the MTX-treated animals could be related to the direct effect of this immunosuppressant on the immune response, but perhaps also to the capacity of the parasites to develop resistance to MTX (29), or because of cross-resistance with Glucantime^®^ (30). This might all be related to the plasticity of the parasites to respond to pharmacological pressure. The fact that *Leishmania* can develop resistance to antimonials and MTX independently (29, 31) raises the question as to whether these resistances can develop at the same time. This will need to be studied further if we are to improve the clinical management of patients. By contrast, the reduced spleen parasite load also found at W6 in the anti-TNF-treated animals was not due to a leishmanicidal effect of this immunosuppressant, but rather a consequence of their use. Blocking TNF cytokine impeded the natural resolution of the infection in the liver (as the immune response is organ specific), which is mediated by granuloma formation (32), explaining the highest parasite load found in this organ at W6. Thus, there was an alteration of the natural course of the infection by the use of anti-TNF immunosuppressant, leading to a delay in the spleen dysfunction.

T cells play a pivotal role in the defence of intracellular infections. A reduction in their numbers is a sign of progression towards VL, while their reestablishment is a marker of recovery (33). In addition, immunosuppressive treatments can cause leucopoenia as a side effect, increasing the severity of any infection (34, 35). In the present work, the immunosuppressed infected animals had the lowest CD4^+^ T levels at W6, which seemed to be related to the progress and severity of *Leishmania* infection. After Glucantime^®^ treatment there were also important differences between the immunosuppressed animals and the control group in terms of circulating T lymphocytes. CD4^+^ T cell levels were re-established in the control group after treatment, but not in the immunosuppressed animals. However, no reestablishment of the CD8^+^ T circulating levels was observed in any of the experimental groups, perhaps as a consequence to the disease itself rather than an effect of immunosuppression. In fact, in patients treated with pentavalent antimonials, CD8^+^ T levels have been reported to slowly increase for up to 6 months after treatment (33, 36).

Hypergammaglobulinemia is a sign of severe VL both in patients and experimental animal models. In mice, a reduction in the antibody titre is associated with a protective Th1-type response, especially if the humoral response is polarized towards the IgG2a subclass rather than IgG1 (37). In the present work, the number of B cells, and the production of antibodies, was increased during VL infection in the control and anti-TNF groups, polarizing the immune responses towards the Th2 type (38). As previously described (17), the blockade of TNF potentiates Th2 and humoral responses, resulting in an increase in the production of IgG antibodies, especially of the IgG1 subtype. Although MTX produced a slightly increased in the number of circulating B lymphocytes after infection, this immunosuppressant seems to avoid the humoral immune response (both expansion of B-cell population and IgG production) in comparison with the control group. Glucantime^®^ treatment was only effective in reducing the number of B cells in the immunocompetent group. It has been reported that B cell-deficient mice have a resistant phenotype, with a stronger Th1-type immune response against *L. donovani* infection compared to wild type mice (39). This might be related to a better response to antimonial treatment in the non-immunosuppressed group of the present study. The B cells of the anti-TNF-treated animals also showed the greatest IL-10^+^ and PD-1^+^ production after SLA stimulation. The increased expression of the exhaustion marker PD-1^+^ by these cells may affect the pro-inflammatory activity of T lymphocytes, increasing the production of *Leishmania*-specific antibodies and contributing to higher parasite loads (40). Moreover, the production of IL-10^+^ by B cells stimulates T lymphocytes to express IL-10^+^ and PD-1^+^ (40, 41). The expression of both exhaustion markers by the B cells of the mice treated with anti-TNF might be associated with the deregulation of the protective immune response and with the progression towards severe VL in the immunosuppressed animals. This needs to be explored in human patients with VL treated with immunosuppressant drugs.

Glucantime^®^ treatment drastically reduces parasite loads, which stimulates the immune system to generate a Th1-type protective response against *Leishmania* (42). However, the weak cellular immune response of immunosuppressed patients considerably reduces the efficacy of antimonials (43, 44). To understand the general cellular immune response of the immunosuppressed mice after Glucantime^®^ treatment, cytokine production was measured in the supernatants of splenocyte cultures stimulated with αCD3/αCD28. The results revealed differences in cytokine secretion depending on the immunosuppressant administered. In the anti-TNF-treated animals, polarization towards a Th2-type profile was seen, with a total absence of the pro-inflammatory cytokines IFN-γ, TNF and IL-2, and an increase in IL-10 and IL-6 basal levels. The inability to develop a Th1 cellular response is associated with parasite persistence and antileishmanial treatment of low efficacy, as observed in the anti-TNF-treated animals. The secretion of both IL-6 and IL-10 is associated with progress towards VL due to the inhibition of TNF expression and the polarization of CD4^+^ T lymphocytes towards the production of anti-inflammatory cytokines (45, 46). In addition, it has been described, both in experimental animal models and in patients with VL, that cytokine IL-17 is associated with protection against *Leishmania* infection and with a rapid response to it (47, 48). In the present work, the anti-TNF group showed lower IL-17 levels than the control group, suggesting a poorer response to Glucantime^®^ treatment. In contrast, the MTX-treated animals showed Th1/Th2-type immune responses since they produced IFN-γ, IL-2, IL-6 and IL-10, which is linked to the greater efficacy of the antiparasitic treatment than that seen in the anti-TNF-treated animals, but more limited than that generated in the non-immunosuppressed controls.

To understand the specific cellular immune response to *Leishmania* after Glucantime^®^ treatment, cytokine production was measured in splenocytes stimulated with SLA. As our group previously described, during the first weeks of *L. infantum* infection differences were noted between the anti-TNF- and MTX-treated animals in terms of the ability of the CD4^+^ and CD8^+^ T cells to produce multiple pro-inflammatory cytokines simultaneously (17). For this reason it was decided that this should also be analyzed after treatment with Glucantime^®^. The results show that the frequency of specific single-producer cells increased in all groups after antimonial treatment. The expected differences in the multi-cytokine production capacity of the T cells after treatment were not seen; in the few studies performed to date in immunosuppressed patients with VL, Glucantime^®^ treatment was seen to gradually increase the number of such T cells from day 60 post-treatment, ensuring more durable immunity over time (36).

In general, the anti-TNF-treated mice had the smallest cDCs population, perhaps indicating that this immunosuppressant induces limited antigen presentation and a lesser activation of the T lymphocytes [these cDCs are mainly responsible for CD4^+^ and CD8^+^ T lymphocyte activation (9, 49)]. Tip-DCs are inflammatory cells involved in the production of the pro-inflammatory molecules TNF and NO (50, 51). The lower frequencies of Tip-DCs observed in the immunosuppressed animals may be linked to their diminished pro-inflammatory responses, which can affect the memory immune response after Glucantime^®^ treatment. Since a direct effect of antimonial treatment is to increase DCs populations, which are important for a long-term immune response, this observation may be related to the poorer response to leishmanicidal treatment under immunosuppressive conditions (52). Moreover, Tip-DCs maintain inflammatory responses and therefore increase the inflammation generated by autoimmune diseases such as psoriasis, making them a specific target of immunosuppressants such as MTX (53). The inhibition of these cells generates a reduced inflammatory response, according to the increased spleen parasite load in MTX-treated animals.

In conclusion, the present results show that immunosuppressive therapy with anti-TNF antibodies or MTX alters the immune response generated in *Leishmania-*infected individuals after Glucantime^®^ treatment. The alterations caused are different, but both lead to more limited reactions than seen in immunocompetent animals. This might explain the greater clinical severity of VL seen in the corresponding experimental mice in this work, and the poorer response to treatment seen in human patients. The fact that pharmacological immunosuppression, especially with anti-TNF, leads to fewer DCs being produced and an immune response polarized towards the *Leishmania*-susceptible profile, might explain the greater risk of relapse in immunosuppressed patients. Given this likely scenario, further studies are needed to improve their clinical management. In fact, similar studies are being carried out with specific mice models of autoimmune diseases to explore these findings in conditions that could mimic better what happened in nature.

## Data availability statement

The original contributions presented in the study are included in the article/supplementary material. Further inquiries can be directed to the corresponding author.

## Ethics statement

The animal study was approved by Committee on Ethics and Animal Welfare of the Instituto de Salud Carlos III. The study was conducted in accordance with the local legislation and institutional requirements.

## Author contributions

LB: Data curation, Formal analysis, Investigation, Writing – original draft, Writing – review & editing. JCS: Conceptualization, Data curation, Formal analysis, Investigation, Writing – original draft, Writing – review & editing. CS: Formal analysis, Investigation, Methodology, Writing – original draft. AT: Formal analysis, Investigation, Methodology, Writing – original draft. ER: Investigation, Formal analysis, Writing – original draft. EC: Conceptualization, Funding acquisition, Writing – review & editing. JM: Conceptualization, Funding acquisition, Writing – review & editing.
